# NLRP3 Inflammasome and Its Critical Role in Gynecological Disorders and Obstetrical Complications

**DOI:** 10.3389/fimmu.2020.555826

**Published:** 2021-01-28

**Authors:** Xuhui Fang, Yanshi Wang, Yu Zhang, Yelin Li, Joanne Kwak-kim, Li Wu

**Affiliations:** ^1^Center for Reproductive Medicine, Anhui Provincial Hospital affiliated to Anhui Medical University, Hefei, China; ^2^Reproductive Medicine and Immunology, Obstetrics and Gynecology, Clinical Sciences Department, Chicago Medical School, Rosalind Franklin University of Medicine and Science, Vernon Hills, IL, United States; ^3^Center for Cancer Cell Biology, Immunology and Infection Diseases, Chicago Medical School, Rosalind Franklin University of Medicine and Science, North Chicago, IL, United States

**Keywords:** nucleotide-binding oligomerization domain-containing protein-, leucine-rich repeats-, and pyrin domain-containing protein 3 (NLRP3), IL-1β, nuclear factor kappa-B (NF-κB), cervical cancer, preterm birth, recurrent pregnancy loss

## Abstract

Inflammasomes, intracellular, multimeric protein complexes, are assembled when damage signals stimulate nucleotide-binding oligomerization domain receptors (NLRs). Several inflammasomes have been reported, including the NOD-, LRR- and pyrin domain-containing protein 3 (NLRP3), NLRP1, NLRP7, ice protease-activating factor (IPAF), absent in melanoma 2 (AIM2) and NLR family CARD domain-containing protein 4 (NLRC4). Among these inflammasomes, the NLRP3 inflammasome is the most well-studied in terms of structure and function. Unlike other inflammasomes that can only be activated by a finite number of pathogenic microorganisms, the NLRP3 inflammasome can be activated by the imbalance of the internal environment and a large number of metabolites. The biochemical function of NLRP3 inflammasome is to activate cysteine-requiring aspartate proteinase-1 (caspase-1), which converts pro-IL-1β and pro-IL-18 into their active forms, namely, IL-1β and IL-18, which are then released into the extracellular space. The well-established, classic role of NLRP3 inflammasome has been implicated in many disorders. In this review, we discuss the current understanding of NLRP3 inflammasome and its critical role in gynecological disorders and obstetrical complications.

## Introduction

Inflammasomes, including NLRP1, NLRP3, NLRP7, IPAF, AIM2, and NLRC4, have been reported to be involved in the pathogenesis of many inflammatory diseases, such as dermatitis, arthritis, interstitial pneumonia, and infantile enterocolitis (1–4). Meanwhile, in the studies of gynecological disorders and obstetrical complications, the NLRP3 inflammasome was found to be highly related to cervical cancer (CC), preterm birth, fetal growth restriction (FGR), recurrent pregnancy losses (RPL), pre-eclampsia (PE), intrauterine fetal death and neonatal hypoxic-ischemic encephalopathy (NHIE) (5–11). Herein, we focus on the NLRP3 inflammasome and its critical role in gynecological disorders and obstetrical complications. The NLRP3 inflammasome is a member of the nucleotide-binding and leucine-rich repeat-containing (NLR) protein family, of which 23 members have been reported in humans (12, 13). The NLRP3 inflammasome is closely related to various heritable and acquired diseases, especially inflammation-driven diseases, such as gout, cardiovascular diseases, type 2 diabetes, Alzheimer’s disease, prion diseases, infectious diseases, gynecological diseases, and obstetrical complications (12, 14–17). This association can be partly explained by mutations and polymorphisms in NLR coding genes. Indeed, multiple NLRP3 gene mutations have been reported in various autoimmune inflammatory diseases, such as cryopyrin-associated periodic syndrome (CAPS), Crohn’s disease, psoriatic juvenile idiopathic arthritis, rheumatoid arthritis, food-induced anaphylaxis, aspirin-induced asthma, urticaria, type 2 DM, hypertension, and cancer. For example, over 90 genetic variants of the NLRP3 gene have been reported in CAPS. Hereditary mutation of the NLRP3 gene is often overlapped with *de novo* mutation, suggesting the presence of hot-spot loci within the NLRP3 gene that exhibits high mutation susceptibility (18).

There are three critical components of NLRP3 inflammasome: NOD-, LRR- and pyrin domain-containing protein 3 (NLRP3), apoptosis-associated speck-like protein containing a caspase recruitment domain (ASC), and caspase-1, which mainly acts as an IL-1β-converting enzyme (12, 19). The activation of NLRP3 inflammasome is triggered by damage-associated molecular patterns (DAMPs) during sterile inflammation or by pathogen-associated molecular patterns (PAMPs) during infections. DAMPs, also called alarmins, are self-originating molecules, including extracellular ATP, ROS, high mobility group box-1 (HMGB1), and uric acid crystals (7, 12, 19–27). PAMPs refer to molecular patterns of pathogens, including bacterial messenger RNA, bacterial DNA, RNA hybrids, bacterial muramyl dipeptide, DNA, viral RNA, fungi, and protozoa (12, 26, 28). Pattern recognition receptors (PRRs) in the membrane or cytoplasm sense the signals of “damage”, then all sorts of changes have occurred in the body to combat danger, and one of the most important is to initiate the assembly of NLRP3 inflammasome (16, 19, 29, 30). In this review, the current understanding of NLRP3 inflammasome, including its activation and regulatory mechanisms, and its possible role in gynecological disorders and obstetrical complications, are documented.

## The Nucleotide-Binding Oligomerization Domain-Containing Protein-, Leucine-Rich Repeats-, and Pyrin Domain-Containing Protein 3 Inflammasome

### The Activation of Nucleotide-Binding Oligomerization Domain-Containing Protein-, Leucine-Rich Repeats-, and Pyrin Domain-Containing Protein 3 Inflammasome; Canonical and Non-Canonical Pathways

Recent studies show that the canonical NLRP3 inflammasome activation in macrophages requires two signals (12, 29). The first signal is priming *via* the nuclear factor Kappa-B (NF-κB)-dependent pathway (29). The priming signal is initiated by various cytokines or PAMPs, such as IL-1β, tumor necrosis factor (TNF) and Toll-like receptor (TLR) ligands (31). These stimulants induce the nuclear translocation of NF-κB by binding to PRRs, such as TLRs, IL-1 receptor, TNF receptor, and nucleotide-binding oligomerization domain-containing protein 2 (NOD2). Following translocation, NF-κB binds to a specific sequence of DNA and causes transcription of various genes involving pro-IL-1β and NLRP3. Finally, this transcription induces the synthesis of pro-IL-1β and the upregulation of NLRP3 at the transcriptional level (12, 24, 29). The priming signal is a complicated process with many participating regulatory factors. For example, FADD and caspase-8 promote the synthesis of pro-IL-1β *via* TLR4 mediated NF-κB activation at the transcriptional level (32–34). The adaptor TIR domain-containing adapter-inducing interferon-β (TRIF) and IRAK1 also regulate the priming signal through IRAK-induced posttranscriptional modification at the posttranscriptional level (24, 35–38). Although the priming signal is an extraordinarily orchestrated process, the time required for this priming is quite short (35, 36). The second signal is activation triggered by various DAMPs or PAMPs, resulting in the formation and activation of the NLRP3 inflammasome. Activation step includes NLRP3 oligomerization, ASC clustering, and caspase-1 recruitment (16). NLRP3 receptor protein contains three domains: pyrin domains (PYD), NACHT (also called the NOD domains) and C-terminal leucine-rich repeats (LRRs) (39, 40). In canonical NLRP3 inflammasome pathway, the signal is triggered by various DAMPs or PAMPs. Then PYD domains of NLRP3 receptor protein interact with PYD domains of ASC, leading to the assembly of ASC (12, 16, 29). Afterwards, CARD domains of ASC interact with CARD domains of the effector protein, caspase domains. As a result, active caspase-1 is released from pro-caspase-1 by self-cutting, resulting in the cleavage of pro-IL-18 and pro-IL-1β (12, 29, 40). Finally, mature and biologically active IL-18 and IL-1β are released, which play significant roles in inflammatory responses (12, 16). Active caspase-1 also induces gasdermin D-mediated programmed cell necrosis, called pyroptosis (19, 30, 41, 42). In pyroptosis, caspase-1 cleaves gasdermin D into gasdermin D^Nterm^. When gasdermin D^Nterm^ is inserted into the cell membrane and forms pores, cell necrosis is induced with the release of IL-1β (38).

Contrarily, non-canonical NLRP3 inflammasome activation is independent on caspase-1. The key activator of the non-canonical NLRP3 inflammasome activation is caspase-11 (12). Most Gram-negative bacteria, including *Escherichia coli*, *Citrobacter rodentium*, and *Vibrio cholerae*, but not Gram-positive bacteria, activate non-canonical NLRP3 inflammasome pathway, suggesting the crucial role of LPS, an immunogenic parietal fragment only from Gram-negative bacteria (30, 43, 44). Due to the CARD domains of caspase-11 bind to the lipid A portion of LPS with high specificity and affinity (44). Meanwhile, although upstream signal of caspase-11 activation is still controversial, type I IFN signaling were supported by several studies (12, 43). Therefore, caspase-11 acts as PRR and senses LPS in the cytoplasm, then resulting in the activation of NLRP3 inflammasome and subsequently IL-1β and IL-18 release (12). And extracellular LPS are recognized by TLR4, then initiating the TRIF signal activation and subsequently nuclear translocation of NF-κB. Afterwards, type I IFN bind to IFN receptor, and JAK/STAT pathway are activated and leading to the transcription of caspase-11 gene. Finally, non-canonical NLRP3 inflammasome activation induce the maturation and release of IL-1β and IL-18, besides pyroptosis (44, 45). Caspase-11-dependent non-canonical NLRP3 activation is independent from canonical NLRP3 activation process (45). In conclusion, the activation of the NLRP3 inflammasome is an extremely complicated process mediated by multiple factors (12, 29, 30).

### The Immune Effects of IL-1β and IL-18

NLRP3 inflammasome is a versatile inflammasome that is capable of reacting to a variety of “damage” signals and inducing the maturation and release of IL-18 and IL-1β (19, 46). Therefore, the inflammasome is considered the most well-characterized molecular platform responsible for IL-18 and IL-1β production (47–49). IL-18, a member of the IL-1 family, is a critical regulator of innate and adaptive immune responses. IL-18 is mainly synthesized by dendritic cells, epithelial cells, and macrophages (50). IL-18 induces the T cell expression of Fas ligand (FasL) and enhances Fas-mediated cytotoxicity. Additionally, IL-18 alone only induces a small amount of IFN-γ and GM-CSF, but in conjunction with IL-12 or IL-15, IL-18 can induce NK cells and CD4 T cells to produce high levels of IFN-γ. Moreover, IL-18 modulates Th2, and Th17 cell responses, and CD8 cytotoxic cell activity by adjusting the microenvironment of the host (51, 52). IL-1β, a member of the IL-1 family, is a potent proinflammatory cytokine that is involved in the majority of the inflammatory reaction (46). IL-1β is mainly synthesized by monocytes and macrophages. IL-1β induces the secretion and release of many cytokines, such as IL-1β itself, IL-1α, IL-6, and TNF-α, and the recruitment of T, B, and NK cells to orchestrate immune responses (50, 53).

### The Activation Pathways of Nucleotide-Binding Oligomerization Domain-Containing Protein-, Leucine-Rich Repeats-, and Pyrin Domain-Containing Protein 3 Inflammasome

Considering that so many stimuli can activate NLRP3 inflammasome, it is unlikely that these stimuli directly interact with the inflammasome. Instead, it is more likely that these stimuli activate the inflammasome through common pathways (12). The pathways that activate NLRP3 inflammasome are still in dispute. However, several hypothetical pathways have been suggested to explain the mechanism of activation (16). These hypotheses include the K^+^ efflux hypothesis, Ca^2+^ mobilization hypothesis, ROS hypothesis, and lysosomal rupture hypothesis. These pathways may be synergistic to a certain extent and not completely exclusive.

**Figure 1:**
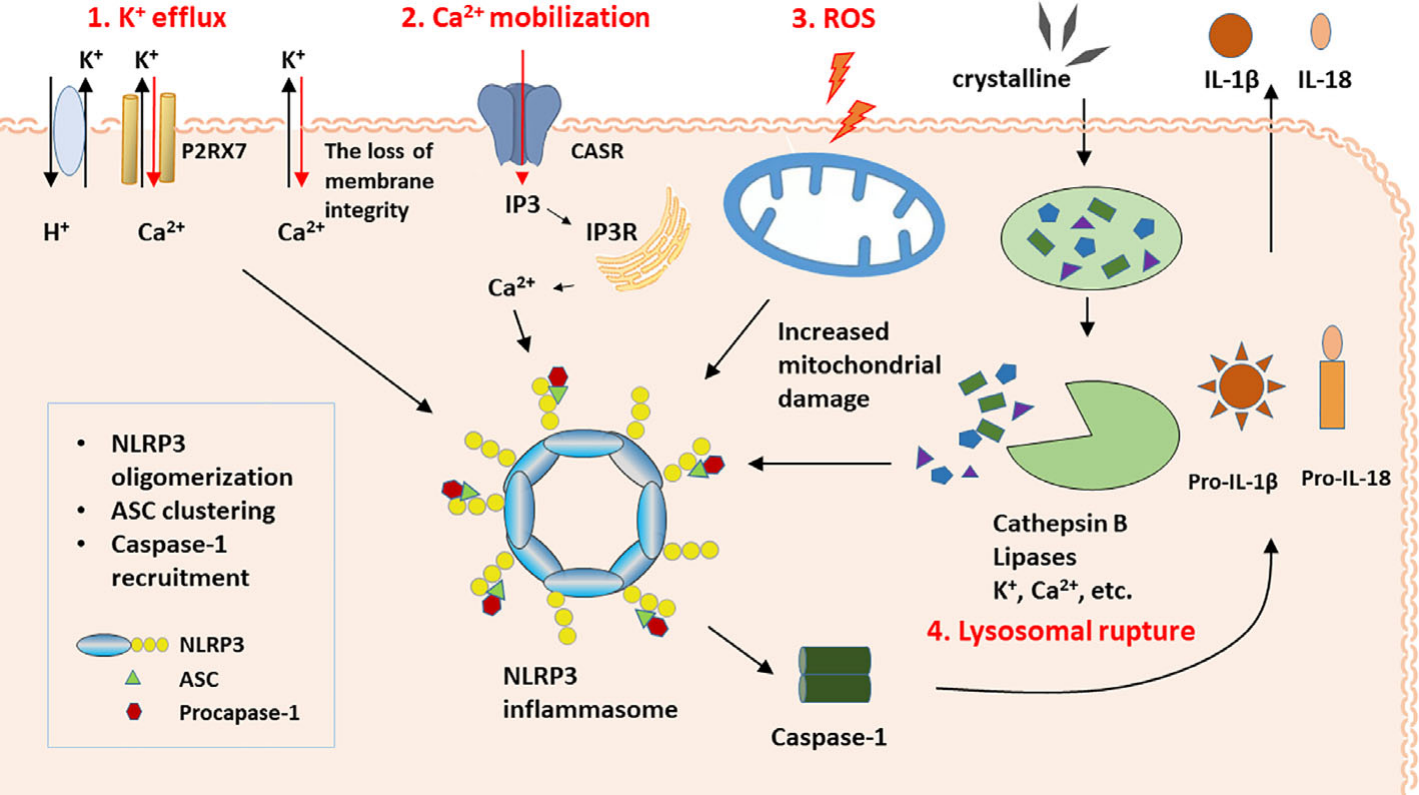
The proposed activation pathways of NLRP3 inflammasome.

When damage signals are transmitted to cells, several consequences may occur. (1) An ATP-gated ion channel named purinergic P2RX7 triggers the efflux of K^+^. In addition, membrane integrity can be destroyed by bacterial pore-forming toxins and the complement membrane attack complex, which may contribute to the K^+^-H^+^ antiporter (16, 54). K^+^ efflux activates the formation of the inflammasome by promoting the never in mitosis gene A-related kinase 7 (NEK7)-NLRP3 interaction, inducing mitochondrial and lysosomal damage, and increasing the production of ROS (55, 56). (2) P2RX7 also triggers the influx of extracellular Ca^2+^. Additionally, an increased influx of extracellular Ca^2+^ into the cytoplasm through the calcium-sensing receptors (CASRs) or damaged cell membranes leads to the activation of phospholipase C (PLC). PLC cleaves phosphatidylinositol 4,5-bisphosphate (PIP2) into DAG and inositol 1,4,5-triphosphate (IP3) *via* PLC-mediated PIP2 hydrolysis. Finally, IP3 binds to its receptor, IP3R, on ER membranes, which triggers Ca^2+^ release (57–59). Ca^2+^ mobilization may activate the inflammasome by promoting NLRP3-ASC complex formation, which acts as a second messenger. Then, this second messenger induces mitochondrial Ca^2+^ overload, followed by the production of ROS and the release of mtDNA and cardiolipin (59–63). (3) Mitochondrial hypoxia, mitochondrial membrane damage, and autophagy/mitophagy inhibition lead to the significant production of ROS (19). (4) Crystalline and particulate matter attack the lysosomal membrane and disrupt the membrane integrity. Then, cathepsin B, the chemical nature of which is a lysosomal cysteine protease, as well as lipases, K^+^, and Ca^2+^ shift their location from the lysosome into the cytoplasm. Finally, cathepsin B, K^+^, Ca^2+^, and activated cell stress-responsive kinases, including TAK1 and JAK, activate NLRP3 inflammasome (12, 16, 29). The proposed pathways are shown in **Figure 1**.

### The Regulators of Nucleotide-Binding Oligomerization Domain-Containing Protein-, Leucine-Rich Repeats-, and Pyrin Domain-Containing Protein 3 Inflammasome

NEK7, as a member of the NIMA-related kinase (NEK proteins) family, is a serine/threonine kinase and is involved in the regulation of the cell cycle and NLRP3 inflammasome (56). The inflammasome response and cell division are exclusive and cannot be carried out at the same time, suggesting that NEK7 may function as a cellular switch (64). The well-established classic role of NEK7 in the activation of NLRP3 inflammasome has been shown in recent studies (56, 64, 65). NEK7 is indispensable for inflammasome activation and is thought to act downstream of K^+^ efflux (66). The high-molecular-mass NLRP3-NEK7 complex is assembled through the interaction of the LRR domain of NLRP3 and the catalytic domain of NEK7 in a kinase-independent manner (64). Schmid-Burgk J.L. et al. reported that NLRP3–NEK7 complex assembly, as well as ASC oligomerization and ASC speck formation, were entirely suppressed when NEK7 was absent (67). Likewise, He Y., et al. observed that caspase-1 activation and IL-1β secretion in response to stimulation by numerous agonists, including ATP, lipopolysaccharide (LPS), nigericin, and alum, were also suppressed without NEK7 (56). Several regulators of NLRP3 inflammasome activation have been reported, such as double-stranded RNA-dependent protein kinase (PKR) and guanylate-binding protein 5 (GBP5); however, the roles of these regulators are still in dispute (68, 69).

### The Agonists and Antagonists of Nucleotide-Binding Oligomerization Domain-Containing Protein-, Leucine-Rich Repeats-, and Pyrin Domain-Containing Protein 3 Inflammasome

Agonists and antagonists activate or inhibit NLRP3 inflammasome through the four pathways described above, either directly or indirectly (26). RIP1 activates NLRP3 inflammasome by forming a complex that promotes mitochondrial damage and ROS production (70). CASR agonists, such as Gd^3+^, AL^3+^, and R-568, and the PLC agonist m-3M3FBS activate NLRP3 inflammasome by promoting Ca^2+^ mobilization (59, 71).

Multiple antagonists for NLRP3 inflammasome have been reported, including indirect inhibitors (glyburide, 16673-34-0, JC124, and FC11A-2), inhibitors for the constituents of NLRP3 inflammasome (parthenolide, VX-740 and -765, BAY 11-7082m and β-hydroxybutyrate) and direct inhibitors of NLRP3 protein (MCC950, 3,4-mehtylenedioxy-β-nitrostyrene, CY-09, tranilast, OLT1177, and oridonin) (72). In addition, RIP2 inhibits inflammasome activation by enhancing autophagy of mitochondria and subsequently reducing ROS (19, 70). CA-074-ME, a cathepsin B inhibitor, blocks inflammasome activation by inhibiting the translocation of cathepsin B from the lysosome to the cytoplasm (16). Currently, none of these molecules is approved by Food and Drug Administration or other governmental agencies. Further development of small molecules with improved therapeutic efficacy is needed.

## Nucleotide-Binding Oligomerization Domain-Containing Protein-, Leucine-Rich Repeats-, and Pyrin Domain-Containing Protein 3 Inflammasome and Clinical Disorders

Increasing numbers of studies indicate that NLRP3 inflammasome is the critical modulator in various inflammatory conditions. When cells and tissues are damaged or dying, DAMPs are released and activate NLRP3 inflammasome and various immune cells, including neutrophils, dendritic cells (DCs) and macrophages (27). Then, these immune effectors release numerous cytokines and chemokines, which in turn recruit more immune cells by inducing the proliferation, differentiation, and migration of immune effectors, subsequently leading to immune activation and sterile inflammation (29). There is an array of DAMPs and DAMP-sensing receptors that participate in sterile inflammation and synergistically orchestrate the initiation, regulation, and termination of sterile inflammation. Sterile inflammation is usually beneficial to the host and promotes the repair and regeneration of cells and tissues (27). However, the protective effect of sterile inflammation is often limited, and sterile inflammation can be detrimental to the body by inducing inflammatory disorders (46, 50).

In type 2 diabetes, chronic hyperglycemia activates NLRP3 inflammasome by promoting the generation of ROS; then, IL-1β induces the dysfunction and destruction of pancreatic islet β cells (16). In gout, monosodium urate (MSU) activates NLRP3 inflammasome, through which IL-1β induces chronic inflammation and inflammatory injuries (73).

### The Possible Molecular Mechanisms of Nucleotide-Binding Oligomerization Domain-Containing Protein-, Leucine-Rich Repeats-, and Pyrin Domain-Containing Protein 3 Inflammasome in Gynecological Disorders and Obstetrical Complications

**Figure 2:**
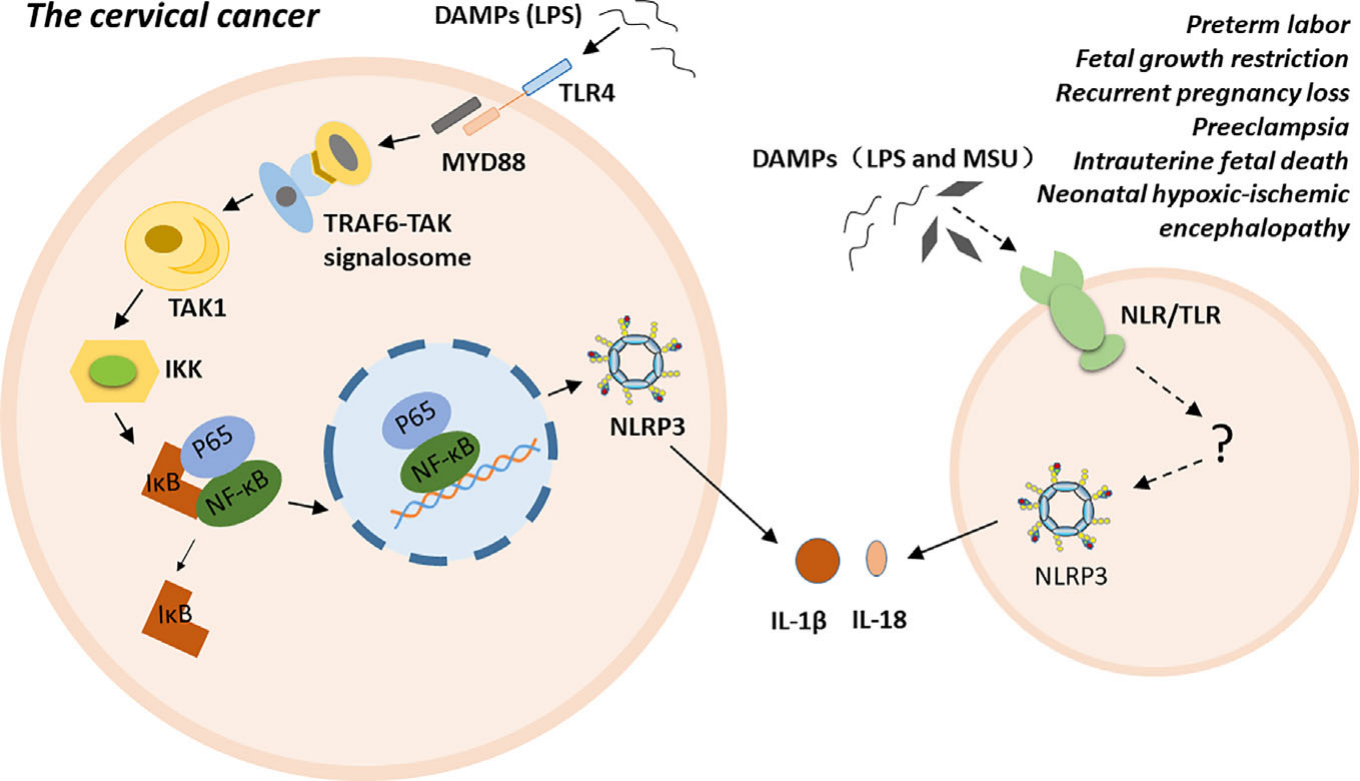
The possible molecular mechanisms of NLRP3 inflammasome in inflammatory conditions.

NLRP3 inflammasome is highly related to multiple gynecological disorders and obstetrical complications, such as cervical cancer (CC), preterm labor, FGR, RPL, PE, intrauterine fetal death, and NHIE (5–11). The possible molecular mechanisms of NLRP3 inflammasome has been proposed in cervical cancer. Particular ligands, such as LPS, trigger carcinogenesis by binding to their receptors, such as Toll-like receptor 4 (TLR4). TLR4, as a membrane PRR, activates the TRAF6-TAK signalosome *via* the myeloid differentiation factor 88 (MyD88)-dependent pathway. Subsequently, the signalosome activates transforming growth factor-β-activated kinase 1 (TAK1) *via* autophosphorylation. TAK1 then phosphorylates IkB kinases (IKKs). The phosphorylated IKKs induce the phosphorylation of inhibitor of nuclear factor kappa-B kinase (IκB), and IκB dissociates from the inactive P65- NF-κB -IκB trimer. The activated P65-NF-κB dimer translocate into the nucleus and triggers the expression, synthesis and release of various proinflammatory cytokines. Finally, persistent inflammation contributes to the malignant transformation of normal cervical cells and the establishment of a tumor microenvironment (74–76). Additionally, polymorphism of the inflammasome component IL-1β is a factor that increases susceptibility to CC at the genetic level (77). For preterm labor, fetal growth restriction, recurrent pregnancy loss, pre-eclampsia, intrauterine fetal death, and neonatal hypoxic-ischemic encephalopathy, the exact mechanism is unclear. Excessive inflammation induced by NLRP3 inflammasome may play a crucial role in the pathogenesis of these conditions either by directly participating in or indirectly regulating various stages of disease progress (**Figure 2**).

### The Role of Nucleotide-Binding Oligomerization Domain-Containing Protein-, Leucine-Rich Repeats-, and Pyrin Domain-Containing Protein 3 Inflammasome in Gynecological Diseases

NLRP3 inflammasome is closely associated with various gynecological diseases, especially in gynecological-oncology conditions (76, 78). Accumulating studies have demonstrated that inflammasomes play a pivotal role in carcinogenesis by recruiting various immune cells, including neutrophils, dendritic cells, natural killer (NK) cells, macrophages, T and B lymphocytes, and inducing inflammatory responses (79–81). Moderate inflammation may contribute to fighting cancer. For example, IL-18 contributes to repairing the epithelial barrier and inducing NK cells to kill tumor cells. Additionally, IL-1β drives the efficient CD8+ T cell response against tumor cells (82, 83). However, persistent inflammation is related to the dysregulation of cell differentiation, angiogenesis, apoptosis evasion, and malignant transformation and progression. Additionally, inflammatory factors may contribute to carcinogenesis by participating in the interaction between cancer cells and the microenvironment (84).

Cervical cancer (CC) is an extraordinarily common cancer in women, second only to breast cancer (85). Studies have shown that chronic inflammation, along with human papillomavirus (HPV) infection, induces carcinogenesis of the cervix (86, 87). However, in a study of women with CC (N=74), the IL-18 expression in cervical tissue was significantly lower in CC groups compared to that of high grade squamous intraepithelial lesion (HSIL) group. In addition, the IL-1β expression was significantly decreased in the CC group compared with those of normal and low grade squamous intraepithelial lesion groups (LSIL) (5). Therefore, a complex immunological mechanism regulates inflammatory cytokine expressions, while the preneoplastic cervical lesions progress to a more advanced state. Further studies are needed to investigate a possible regulatory role of the inflammasomes in inflammatory cytokine production.

In a study using LPS-stimulated human CC cells, human SiHa and Caski cells (HPV-16-infected cervical cancer cell lines), the mRNA and protein levels of inflammasome components, such as NLRP3, pro-IL-1β, IL-1β, and caspase-1, was significantly increased. When immunosuppressor CD200Fc was added, in addition to IL-1β, caspase-1, NLRP3, ASC, the protein levels of TLR4 and P65, and the translocation of P65 to the nucleus were significantly decreased in a dose-dependent manner, suggesting the crucial role of CD200Fc in the modulation of TLR4- NF-κB and NLRP3 inflammasome pathway. However, in HeLa cells (HPV18-infected cervical cancer cell line) and C33A cells (HPV-negative cervical cancer cells), no such changes were observed (76). In HeLa cells and C33A cells, the TLR4 mRNA level was found no significant difference when treated with different doses of LPS, suggesting HeLa cells and C33A cells did not have an obvious response to LPS (86) In HeLa cells and C33A cells, the TLR4 mRNA level was found no significant difference when treated with different doses of LPS, suggesting HeLa cells and C33A cells did not have an obvious response to LPS (86).

### The Role of Nucleotide-Binding Oligomerization Domain-Containing Protein-, Leucine-Rich Repeats-, and Pyrin Domain-Containing Protein 3 Inflammasome in Obstetrical Complications

The fetus and placenta, as a semiallograft, express not only maternal antigens but also paternal antigens (9). Therefore, pregnancy presents a unique immune challenge to the mother, and maternal-fetal immunotolerance is essential for a successful pregnancy. Thus, the bias toward immune tolerance is one of the determinants of a successful pregnancy. During normal pregnancy, the maternal adaptive immune system is inhibited, and the innate immune system is relatively activated. These changes have been reported to be beneficial for a successful pregnancy (88). In pregnant women, the inflammasome is mainly assembled in the placenta, which is a vital organ that is responsible for successful pregnancy (46, 89, 90). The placenta regulates its function and the progress of the pregnancy by producing pro- and anti-inflammatory cytokines, including IL-1β, IL-6, TNF-α (91, 92). Inflammatory dysfunction of the placenta may contribute to the dysregulation of immune responses at the maternal-fetal interface and result in devastating consequences (10, 20, 89). Excessive placental inflammation directly or indirectly affects the mother and the offspring, and it is highly associated with a number of specific obstetrical complications, such as preterm birth, FGR, RPL, PE, intrauterine fetal death and NHIE (6–11). NLRP3 inflammasome, as a critical constituent of the immune system, is intimately involved in the inflammatory response of these conditions (88, 90, 93, 94).

#### Preterm Birth

Preterm birth is defined as delivery occurring before 37 weeks of gestation. Preterm birth is the primary cause of neonatal morbidity and mortality worldwide, and the incidence is reported to be 10% or higher (95). As a syndrome that results from multiple etiologies, a close association between NLRP3 inflammasome and preterm birth has been reported (6, 17). NLRP3 inflammasome can be activated by amniotic infection, leading to the initiation of labor, membrane rupture, and cervical dilatation, finally resulting in preterm birth (6, 96).

In a study of women with preterm births (N=37), membranes with acute chorioamnionitis had increased levels of HMGB1, caspase-1, IL-18, and IL-1β compared with those from women who underwent normal labor (17).

In LPS-induced C57BL/6 mice preterm birth model, the mRNA and protein levels of NLRP3, caspase-1, IL-18, and IL-1β in the fetal membrane and basal decidua were markedly elevated as compared with those of the phosphate-buffered saline (PBS)-treated control mice, suggesting that NLRP3 inflammasome may play a critical role in proinflammatory changes. Moreover, when mice undergoing LPS-induced preterm birth were treated with the inflammasome inhibitor MCC950, the time interval from the LPS injection to delivery was prolonged, and the preterm birth rate was reduced by 30%. Additionally, the neonatal mortality rate was decreased by approximately 30% (97). These findings were consistent with the previous study, which reported a 50% preterm birth rate when the alarmin S100B was injected into the C57BL/6 mouse model, whereas MCC950 reduced the preterm birth rate and neonatal morbidity by 35.7% and 26.7%, respectively (6).

#### Fetal Growth Restriction

FGR is defined as a fetal weight below the tenth percentile of normal fetal weights (98). Excessive placental inflammation was reported to be associated with FGR.

In the MSU-induced FGR rodent model, the prevalence of FGR was increased in a dose-dependent manner. 20% of pups developed growth restriction when the pregnant rat was treated with MSU 250 mcg/Kg. When pregnant rats were treated with MSU 500 or 1000 mcg/Kg, over 80% fetal growth restriction was observed in comparison to the PBS treated controls. Additionally, the growth restriction induced by MSU was reversed by a caspase-1 inhibitor or IL-1β inhibitor (7).

It is speculated that in pathological pregnancies, increases in various DAMPs from the maternal-fetal interface, such as increased levels of MSU and HMGB1, trigger the activation of the NLRP3 inflammasome, which in turn increases IL-1β. Increased IL-1β may induce defective spiral artery remodeling, aberrant uteroplacental hemodynamics, decreased system A amino acid transporter activity, cytotrophoblast cell apoptosis, and decreased syncytialization. All of these pathological changes may conspire to the development of FGR (7, 99, 100).

#### Recurrent Pregnancy Loss

RPL, defined as two or more consecutive clinical pregnancy losses prior to 20 weeks of gestation, is the most common pregnancy complication, and approximately 5–15% of all pregnancies can be affected (101). Of all the causes leading to RPL, NLRP3 inflammasome has been demonstrated to play an important role in RPL by establishing inflammatory responses during pregnancy (102).

In a study of endometrial tissues derived from women with RPL (N=30), the endometrial expression of NLRP3, caspase-1, IL-18, and IL-1β was significantly increased compared with that of normal fertile women (46). In another study of endometrial tissues obtained from women with RPL (N=36), increased secretion of proinflammatory cytokines, such as IL-1β, TNF-α, and IFN-γ was reported. In contrast, the secretion of anti-inflammatory cytokines, such as IL-4 and IL-10, and angiogenic cytokines, including IL-2, IL-6, and IL-8, was reduced compared with those of normal fertile women (103). Mutations in NLRP7 and KHDC3L were known to cause familial biparental hydatidiform moles (BiHMs), a rare form of pregnancy loss and endometrial cancer. However, the study did not find any relationship between NLRP7, NLRP2, KHDC3L, and RPL (104).

It is generally accepted that the establishment and maintenance of a successful pregnancy rely on a balance between pro- and anti-inflammatory responses at the maternal-fetal interface. Additionally, endometrial cells convert into a specific phenotype that is suitable for embryo implantation, subsequent placenta and fetal development, and spiral artery invasion, which often requires inflammatory cytokines (105). Women with RPL have a higher incidence of abnormal intestinal permeability and increased plasma LPS levels. The impaired intestinal epithelial barrier may increase intestinal permeability, which permits the entry of Gram-negative bacteria into the bloodstream. Then, LPS, an immunogenic parietal fragment from bacteria, is produced. Subsequently, large amounts of inflammatory cytokines can be produced. Therefore, NLRP3 may be associated with endometrial proinflammatory micro-milieu and defective angiogenesis, which contribute to RPL by disturbing the inflammatory balance at the maternal-fetal interface (7, 104, 106, 107).

#### Pre-Eclampsia

PE, characterized by the presence of increased blood pressure (BP>140/90 mmHg) and proteinuria (>300 mg/24 h) after 20 weeks of gestation, is usually accompanied by headache, nausea, vomiting, and epigastric discomfort (108, 109). PE is a multisystem pregnancy disorder that causes severe maternal symptoms (88). Among the factors that contribute to PE, including impaired spiral artery remodeling, endothelial dysfunction, and excessive maternal systemic inflammation, the systemic inflammation accounts for many of the remaining factors (110–112).

In a study of women with PE (N=20), Ingrid C Weel et al. reported that the placental expression of NLRP3, IL-1β, and caspase-1 was significantly increased in the placenta from PE women compared to that from normotensive pregnant women, indicating that NLRP3 inflammasome may participate in the development of PE (9).

In a mice model, C57BL/6 mice were injected with extracellular vesicles (EV), which induces the accumulation of activated platelets in the placental bed and activates the NLRP3 inflammasome. EV-injection induced PE-like phenotypes with renal dysfunction and elevated blood pressure. In addition, the expression of NLRP3, caspase-1, and IL-1β was increased in the placentas of EV-injected mice compared to those of control mice. Additionally, when NLRP3/caspase-1 knockout mice or mice given NLRP3 inflammasome inhibitors received the same treatment, the PE-like phenotype did not appear (113).

#### Neonatal Hypoxic–Ischemic Encephalopathy

NHIE is associated with 23–25% of neonatal death worldwide and results in long term sequelae if affected neonates survive (114, 115). Maternal hypotension, cardiac arrest, placental thrombosis, placental abruptio, uterine rupture, and placental inflammation have been reported to be the risk factors for NHIE. In the brain parenchyma, hypoxia-ischemia has been demonstrated to induce inflammatory responses, followed by subsequent neuronal death mediated by the peripheral immune system (116, 117).

Activated microglial cells release proinflammatory cytokines and ROS, and microglial inflammation is reported to be regulated by the NLRP3 inflammasome (118). In an experimental chronic migraine mice model, NLRP3 and IL-1β were mainly expressed in the microglia in the trigeminal nucleus caudalis (TNC), while IL-1R was expressed in the neurons. Activation of NLRP3 inflammasome in microglia mediates IL-1β release by promoting the process of pro-IL-1β to mature one (48, 119). In a preclinical rodent model of LPS+Hypoxia/ischemia-induced encephalopathy, neuronal IL-1β upregulated cytokine-induced neutrophil chemoattractant, monocyte chemoattractant protein -1 and inducible nitric oxide synthase, suggesting its role in PMN infiltration. IL-1βwas also associated with the activation of an apoptotic and necroptotic pathway by increasing the expression of activated caspase-3 and receptor-interacting-protein-3 (120). In a rodent model undergoing hypercapnia/hypoxemia, cognitive impairment, apoptosis of hippocampal neurons, NLRP3 inflammasome activation, and upregulation of IL-1β had interaction effects. In hypoxia-activated microglia, the expression of NLRP3, caspase-1, and IL-1β was significantly increased by hypercapnia (121).

In a cord blood study of women undergoing cesarean section and vaginal delivery, IL-1β level was significantly lower in elective cesarean section cases as compared with those of emergency cesarean section and vaginal delivery, suggesting cord blood IL-1β level may determine high-risk babies for perinatal hypoxic stress (122). The NLRP3 inflammasome in neonates with NHIE has not been elucidated well, and more studies are needed in the future.

## Conclusion

In this review, the components, assembly, and activation of NLRP3 inflammasome are discussed. Although multiple hypotheses, such as the K^+^ efflux, Ca^2+^ mobilization, ROS production, and lysosomal rupture hypotheses, have been proposed to explain inflammasome activation, a widely accepted theory has not yet emerged. In addition, NLRP3 inflammasome has been indicated to play an important role in the pathogenesis of various gynecological and obstetrical diseases. Therefore, NLRP3 inflammasome might be an attractive therapeutic and diagnosis target NLRP3 inflammasome-related disorders. However, clinical studies of NLRP3 inflammasome in normal pregnant women, women with gynecological and obstetrical complications, and neonates with NHIE are significantly limited. Further clinical studies and studies to investigate the feasibility and safety of targeted therapy are needed.

## Author Contributions

All authors who participated in the work are listed and agreed for publication. All authors contributed to the article and approved the submitted version.

## Funding

This study was supported by the National Natural Science Foundation of China (82071650) and (82001641).

## Conflict of Interest

The authors declare that the research was conducted in the absence of any commercial or financial relationships that could be construed as a potential conflict of interest.
